# Metformin and 4SC‐202 synergistically promote intrinsic cell apoptosis by accelerating ΔNp63 ubiquitination and degradation in oral squamous cell carcinoma

**DOI:** 10.1002/cam4.2206

**Published:** 2019-04-25

**Authors:** Yuan He, Shanshan Tai, Miao Deng, Zhaona Fan, Fan Ping, Lihong He, Chi Zhang, Yulei Huang, Bin Cheng, Juan Xia

**Affiliations:** ^1^ Department of Oral Medicine Guanghua School of Stomatology, Sun Yat‐sen University Guangzhou P.R. China; ^2^ Guangdong Provincial Key Laboratory of Stomatology Guanghua School of Stomatology, Sun Yat‐sen University Guangzhou P.R. China

**Keywords:** 4SC‐202, apoptosis, metformin, oral squamous cell carcinoma, ΔNp63

## Abstract

Oral squamous cell carcinoma (OSCC) is the most common and aggressive epithelial tumor in the head and neck region with a rising incidence. Despite the advances in basic science and clinical research, the overall survival rate of OSCC remains low. Thus finding novel effective therapeutic agents for OSCC is necessary. In this study, we investigated the effects and mechanisms of combined metformin and 4SC‐202 in OSCC. Our results showed that metformin and 4SC‐202 synergistically suppressed the proliferation and promoted the intrinsic apoptosis of OSCC cells in vitro and in vivo. Importantly, the proteasome inhibitor MG132 impeded the ΔNp63‐decreasing effects after metformin and 4SC‐202 treatment, indicating that metformin and 4SC‐202 could promote the degradation of ΔNp63 protein. Moreover, ubiquitination level of ΔNp63 increased after metformin or/and 4SC‐202 administration. Furthermore, we revealed that ΔNp63 mediated anticancer effects of metformin and 4SC‐202, as overexpression or suppression of ΔNp63 could attenuate or facilitate the apoptosis rate of OSCC under metformin or/and 4SC‐202 treatment. Collectively, metformin and 4SC‐202 synergistically promote intrinsic apoptosis through accelerating ubiquitin‐mediated degradation of ΔNp63 in OSCC, and this co‐treatment can serve as a potential therapeutic scheme for OSCC.

## 1 INTRODUCTION

Oral squamous cell carcinoma (OSCC) is the most common cancer of oral cavity, and it accounts for more than 90% of all oral tumors.^1^ OSCC is a highly malignant tumor with a delayed clinical detection and poor prognosis.^2^ Current therapeutic strategies for OSCC mainly include surgery, radiation therapy, and chemotherapy. However, despite advances in therapeutic strategies, survival rates of OSCC have not improved considerably in recent years. Therefore, it is necessary to identify novel effective therapeutic agents for OSCC treatment.

Protein acetylation modification plays a vital role in the epigenetic regulation of gene expression. Acetylation of histone generally results in activation of gene; however, deacetylation catalyzed by histone deacetylase (HDAC) results in chromatin condensation and downregulation of gene expression.^3^ Imbalance in the acetylation and deacetylation is responsible for the development and progression of wide variety of cancer.^4^, ^5^ In OSCC, high expression of HDACs, such as HDAC1, HDAC2, HDAC6, was shown to associate poor prognosis, advanced stage, larger tumor size, and lymph node metastasis in patients,^6^, ^7^, ^8^ indicating that HDACs plays vital role in OSCC progress and could be the potential therapeutic target. Histone deacetylase inhibitors (HDACis) increase the level of acetylated lysine residues of core histone which in turn reactivates the expression of silenced genes in the cancerous cell.^9^ Histone deacetylase inhibitors such as suberoylanilide hydroxamic acid, apicidin, panobinostat, and valproic acid could inhibit the growth and induce the apoptosis in head and neck squamous cell carcinomas (HNSCC).^10^, ^11^, ^12^, ^13^ Moreover, as combined administration of chemotherapeutics could take the advantage of each drug, further evaluation of HDAC inhibitors in combination with other chemotherapeutics or potential chemotherapy drug in HNSCC may be justified.

Metformin, a low cost antidiabetic drug, has been widely used to treat diabetes by inhibiting hepatic gluconeogenesis and enhancing glucose uptake in skeletal muscle.^14^ Several studies have revealed that metformin treatment to diabetic patients was associated with lower cancer incidence.^15^, ^16^, ^17^ Furthermore, metformin was repurposed as anticancer therapeutics for different types of cancers such as breast cancer, ovarian cancer, prostate cancer, bladder cancer and HNSCC cells with low toxicity.^18^, ^19^, ^20^, ^21^, ^22^ Intriguingly, several studies have demonstrated that metformin can increase oral cancer cell sensitivity to chemotherapeutic drugs such as 5‐FU, gefitinib, improve treatment efficacy and lower doses and toxicity.^23^, ^24^ Collectively, metformin combined with other chemotherapeutics could be a potential candidate for the development of new treatment strategies for human OSCC. 4SC‐202 is a novel selective class I histone deacetylase inhibitor. In vitro, 4SC‐202 was found to inhibit survival and proliferation of several type of cancer cells including hepatocellular carcinoma cell, colorectal cancer cell, medulloblastoma cell, and urothelial carcinoma cell;^25^, ^26^, ^27^, ^28^ and phase I clinical trials for the treatment of hematological malignant tumor revealed that 4SC‐202 is safe, well tolerated with signs of antitumor activity.^29^ Thus, 4SC‐202 seems to be a promising treatment strategy for oral cancer.

In this study, we evaluated the efficacy and mechanism of combined therapy with metformin and 4SC‐202 in OSCC. Here, we found that metformin and 4SC‐202 synergistically inhibited growth of OSCC in vitro and in vivo. Importantly, our results revealed that combined metformin and 4SC‐202 treatment promoted intrinsic apoptosis by accelerating ΔNp63 ubiquitination and degradation in OSCC. These findings highlighted combined treatment of metformin and 4SC‐202 as a promising potential therapeutic strategy for OSCC.

## 2 MATERIALS AND METHODS

### 2.1 Cell lines and cell culture

Human OSCC cell lines HSC6 were kindly provided by J. Silvio Gutkind (NIH, Bethesda, MD), and HSC3 was obtained from professor Qianming Chen (State Key Laboratory of Oral Diseases, Sichuan University, China). The cells had been tested and authenticated by DNA (STR) profiling. The HSC3 and HSC6 cells were cultured in Dulbecco's Modified Eagle's Medium (DMEM, Gibco, Grand Island, NY) supplemented with 10% fetal bovine serum (FBS, Gibco). All cells were cultured at 37°C in a humidified atmosphere containing 5% CO_2_.

Information regarding reagents and antibodies are listed in Table S1.

### 2.2 Cell proliferation assay

Cell proliferation was determined by the cell counting kit‐8 (CCK‐8, Dojindo, Kumamoto, Japan). Briefly, 2 × 10^3^ cells were seeded into 96‐well plates, and then treated with different concentration of metformin or/and 4SC‐202 for 24, 48, and 72 hours, respectively. The absorbance was measured at 450 nm using a microplate reader (Genios TECAN, Männedorf, Schweiz). All experiments were performed in triplicate. The percentage of cell survival was calculated as follows: cell viability = OD (treated cells)/OD (control cells)  100%.

The IC_50_ values of the two cancer cell lines were calculated using sigmoidal dose response curve‐fitting models (Graphpad Prism, La Jolla, CA). The effects of combination were estimated using the CalcuSyn software (Biosoft, Cambridge, UK). The combination index (CI) was the ratio of the combination dose to the sum of the single‐agent doses at an isoeffective level. A CI value less than 1.0 indicates synergy, and a CI value equal to 1.0 defines additivity, whereas a CI value larger than 1.0 shows antagonism.

### 2.3 Colony formation assay

For colony formation assays, 5 × 10^2^ cells were seeded into 6‐well plates, and 24 hours later they were treated with 0.4 μmol/L 4SC‐202 or/and 16 mmol/L metformin. Colonies cultured for 10 days were visible and then stained with crystal violet. Colonies with diameters above 1 mm were counted.

### 2.4 Xenograft model

A total of 24 female BALB/c nude mice (Laboratory Animal Center of Sun Yat‐sen University, Guangzhou, China), 4‐6‐week‐old and weighing 14 to 16 g, were divided into four groups (control, metformin, 4SC‐202, and combination, n = 6). For subcutaneous injections, 6 × 10^6^ HSC6 cells were injected into the right forelimb of each nude mice. Tumor volume (mm^3^) was measured every 4 days by vernier calipers and calculated by the following formula: V = L × W^2^/2, where L represents the length and W represents the width. Then the mice were sacrificed, and the tumors were collected and weighed at the end of 25 days after injection. The livers and kidneys were collected at the same time.

All the animal procedures were conducted in accordance with the Guidelines for the Care and Use of Laboratory Animals and were approved by the Institutional Animal Care and Use Committee at Sun Yat‐sen University.

### 2.5 4NQO‐induced oral carcinogenesis mice model

A total of 24 female C57BL/6 mice (Nanjing Biomedical Research Institute of Nanjing University, Nanjing, China), 6‐week‐old and weighing 16 to 18 g, were divided into three groups (control, combination, and cisplatin, n = 8). Mice were fed daily with 50 μg/mL 4‐nitroquinoline 1‐oxide (4NQO, Sigma‐Aldrich, Germany) in their drinking water for 16 weeks, and then fed with distilled water for an additional 6 weeks. Fresh 4NQO or water was supplied every week. At week 18, mice with visible lesions of tongue dysplasia were treated with different agents, solvent as negative control, metformin (100 mg/kg, intraperitoneal injection) and 4SC‐202（80 mg/kg, intragastric administration), cisplatin (1 mg/kg, intraperitoneal injection) as positive control for 4 weeks. All animals were euthanized on week 22, and tissue retrieval was done as described previously. All animals were monitored daily for general behavioral abnormalities, signs of toxicity, illness, or discomfort.

All the animal procedures were conducted in accordance with the Guidelines for the Care and Use of Laboratory Animals and were approved by the Institutional Animal Care and Use Committee at Sun Yat‐sen University.

### 2.6 Immunohistochemical assay

Immunohistochemical (IHC) staining was performed according to the manufacturer's instructions. The sections were incubated overnight at 4°C with primary antibodies against ΔNp63 (1:200), visualized using 3, 3‐diaminobenzidine (DAB, Sigma‐Aldrich, Germany) and counterstained with hematoxylin. Two senior pathologists blinded to the data assessed and scored the IHC results. The fields for each sample were randomly selected under a light microscope with a 400 × magnification. The staining index (SI) for ΔNp63 was scored according to the staining intensity (0, no staining; 1, weak, light yellow; 2, moderate, yellow brown; 3, strong, brown) and the proportion of positive cells (0, <5%; 1, 5%‐30%; 2, 30%‐70%; 3, >70%) by the following formula: SI = the proportion of positively stained cells  the staining intensity. Finally, all samples were assigned to three levels according to the S value: negative, S = 0; low expression, 0 < S < 6; high expression, 6 ≤ S ≤ 9.

### 2.7 Cellular apoptosis assay

For apoptosis assay, the Annexin V‐fluorescein isothiocyanate (FITC)/propidium iodide (PI) double‐staining apoptosis detection kit (Roche Diagnostics GmbH, Mannheim, Germany) was used. The OSCC cells were collected and stained with 5 μL Annexin V‐FITC and 5 μL PI after treating with 0.4 μmol/L 4SC‐202 or/and 16 mmol/L metformin for 24 and 48 hours, according to the manufacturer's instructions. The acquisition and analysis of the apoptosis data were performed on a flow cytometer (FACS Calibur, BD Biosciences, USA). Basal apoptosis was determined using the same method in control cells.

### 2.8 Terminal deoxynucleotidyl transferase‐mediated dUTP nick end labeling assay

Terminal deoxynucleotidyl transferase‐mediated dUTP nick end labeling (TUNEL) assays were performed to identify the apoptotic cells using the FragEL™ DNA Fragmentation Detection kit (Calbiochem, EMD Chemicals Inc, Gibbstown, NJ) according to the manufacturer's instructions. Then the sections were stained with DAB solution and counterstained with hematoxylin. For the evaluation of the slides, 100 tumor or epithelial cells were counted per high‐power field (original magnification, 400).

### 2.9 Western blot

The cells were lysed with RIPA buffer supplemented with protease inhibitor (Abcam, Cambridge, MA, UK). BCA protein assay kit (CWbiotech, Beijing, China) was used to quantify the concentrations of the lysates. The lysates were then mixed with loading buffer (4:1; Cwbiotech) and denatured at 99˚C for 5 minutes. The lysates (40 μg/lane) were separated on 10%‐12% sodium dodecyl sulfate polyacrylamide gel electrophoresis (SDS‐PAGE) gels and then transferred onto a PVDF membrane (Millipore, Billerica, MA). Then the membrane was blocked in 5% non‐fat milk for 1 hour at room temperature and incubated with primary antibodies overnight at 4˚C. Subsequently, the membrane was washed in 0.1% TBST for three times and incubated with HRP‐conjugated secondary antibody for 1 hour at room temperature. A highly sensitive chemiluminescence detection system (Millipore) was used to visualize the immunoreactive bands and ImageJ (Bethesda, MD) was utilized to analyze the immunoreactive bands by densitometry. Similar results were obtained from three independent experiments.

### 2.10 Cell transfection

The ΔNp63‐flag plasmid was purchased from Addgene (#26979, Watertown, MA). The ΔNp63‐flag plasmid and the negative control were transfected into OSCC cells by Lipofectamine 3000 (Invitrogen, CA) according to the manufacturer's instructions. The ΔNp63 siRNA (50 nmol/L, RiboBio, Guangzhou, China) and negative controls (NC) were transfected into OSCC cells using the Lipofectamine RNA iMAX Transfection Reagent (Invitrogen) following the manufacturer's instructions. The ΔNP63 sequences were as follows: siΔNp63, 5′‐AGGACAGCAGCATTGATCA‐3′.

### 2.11 Quantitative real‐time PCR

Total RNA was isolated from cells using TRIzol (Invitrogen) and reversed to synthesize cDNA according to the manufacturer's procedure (TaKaRa, Shiga, Japan). The real‐time PCR (RT‐PCR) was carried out using the LightCycler 480 SYBR Green I Master system (Roche, Basel, Switzerland). The relative expression levels were calculated using the 2^−ΔΔCt ^method after normalization to the *GAPDH* expression levels. The primers used were as follows: for *ΔNP63*, forward primer, 5′‐GAAGAAAGGACAGCAGCATTGA‐3′ and reverse primer, 5′‐GGGACTGGTGGACGAGGAG‐3′; and for *GAPDH*, forward primer, 5′‐GCACCGTCAAGGCTGAGAAC‐3′ and reverse primer, 5′‐TGGTGAAGACGCCAGTGGA‐3′.

### 2.12 Co‐immunoprecipitation

HSC3 and HSC6 cells were lysed in low‐salt buffer (20 mmol/L Tris‐HCl, pH 8; 137 mmol/L NaCl; 2 mmol/L EDTA; 1% NP40) supplemented with protease inhibitor cocktail (Abcam). The total protein concentrations were measured by the BCA protein assay kit. Later, the equivalent protein lysates were immunoprecipitated as described above with appropriate ΔNp63 antibody or IgG antibody incubated overnight at 4°C, and then with 40 μL of protein A/G‐Agarose mix (Millipore) at 4°C for 16 hours with gentle rotation. Immunoprecipitates were washed three times with wash buffer and subjected to SDS‐PAGE electrophoresis, then were detected with ubiquitin antibodies.

### 2.13 Statistical analysis

All statistical analyses were undertaken with SPSS 20.0 software (SPSS, Chicago, IL) or the GraphPad Prism 6.0 software (La Jolla, CA). All results shown represent the means ± standard deviation from triplicate experiments performed in a parallel manner, unless otherwise indicated. Statistical analyses were performed using one‐way ANOVA or Krukcal‐Wallis test, where appropriate. A two‐tailed value of *P* < 0.05 was considered statistically significant.

## 3 RESULTS

### 3.1 Metformin and 4SC‐202 synergistically suppressed OSCC proliferation and colony formation in vitro and in vivo

**Figure 1:**
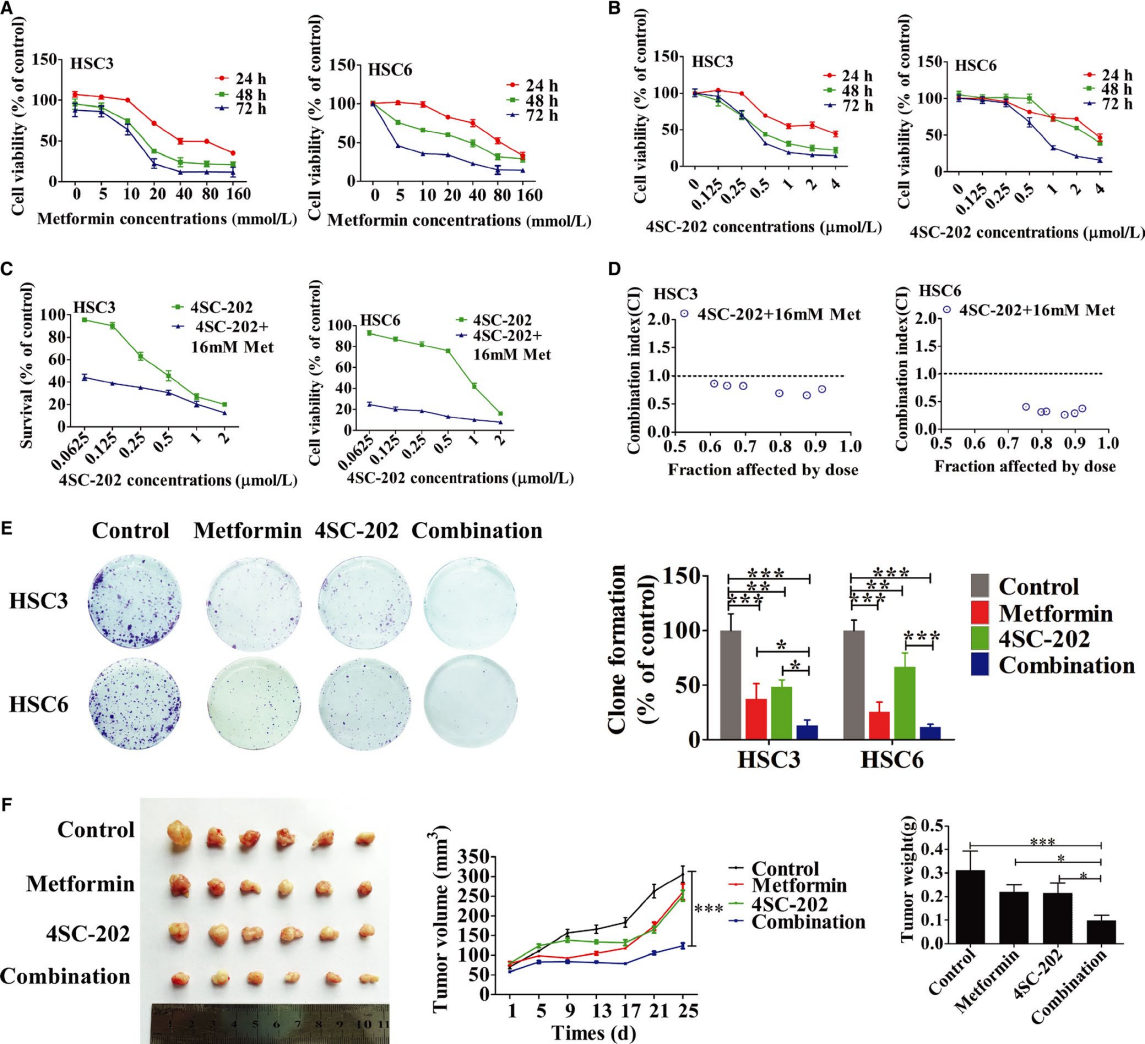
Metformin and 4SC‐202 synergistically inhibited tumor growth in vitro and in vivo. (A‐B) HSC3 or HSC6 were treated with different concentration of metformin or/and 4SC‐202 for 24, 48, and 72 hours, respectively, then CCK8 was used to determine the cell viability. (C) HSC3 or HSC6 were treated with metformin (16 mmol/L) combined with different concentration of 4SC‐202 for 72 hours. (D) The combination effect of metformin and 4SC‐202 in HSC3 and HSC6. (CI < 1 means synergistic effect; CI > 1 means antagonistic effect; CI = 1 means additive effect.). (E) OSCC cells were incubated with 0.4 μmol/L 4SC‐202 or/and 16 mmol/L metformin for 10 days, then colony formation assay was performed. Representative image of colony formation assay was shown. (F) Nude xenograft model was used. Nude mice received injection of HSC6 cells and was treated with metformin (100 mg/kg) or/and 4SC‐202 (80 mg/kg) for 25 days, then tumor volume was measured and weighed. Data were shown as the means ± SD from three independent experiments. ^*^ *P* < 0.05, ^**^ *P* < 0.01, ^***^ *P* < 0.001 vs control (one‐way ANOVA)

To determine the effects of metformin or 4SC‐202 on cell viability, CCK8 was performed. The results showed that metformin and 4SC‐202 suppress the cell viability of OSCC in a time and concentration‐dependent manner (Figure 1A,B). IC_30__‐__40_ of HSC3 and HSC6 at 24 hours (metformin, 16 mmol/L; 4SC‐202, 0.4 μmol/L) was selected for subsequent experiments in consideration of toxicology. Subsequently, CI index was calculated to determine the combination effects of 4SC‐202 and metformin, which revealed that metformin and 4SC‐202 synergistically suppress OSCC proliferation as CI < 1 (Figure 1C,D). Furthermore, the colony forming efficiency was restrained after metformin and 4SC‐202 treatment (*P* < 0.05) in OSCC when compared with single treatment, especially in combination group (Figure 1E). Nude mice with HSC6 tumor xenografts were used to examine the antitumor activity of 4SC‐202 or/and metformin treatment in vivo. The combination treatment showed significant reduction in tumor volume and tumor weight (*P* < 0.05) compared to single treatment (Figure 1F). In addition, the body weight of mice with metformin or/and 4SC‐202 treatment remained unperturbed compared to control group (Figure S1A), and no obvious pathological alteration in the liver and kidney was observed (Figure S1B). Overall, metformin and 4SC‐202 synergistically suppress the OSCC growth in vitro and in vivo.

### 3.2 Metformin and 4SC‐202 synergistically promoted intrinsic cell apoptosis in OSCC

**Figure 2:**
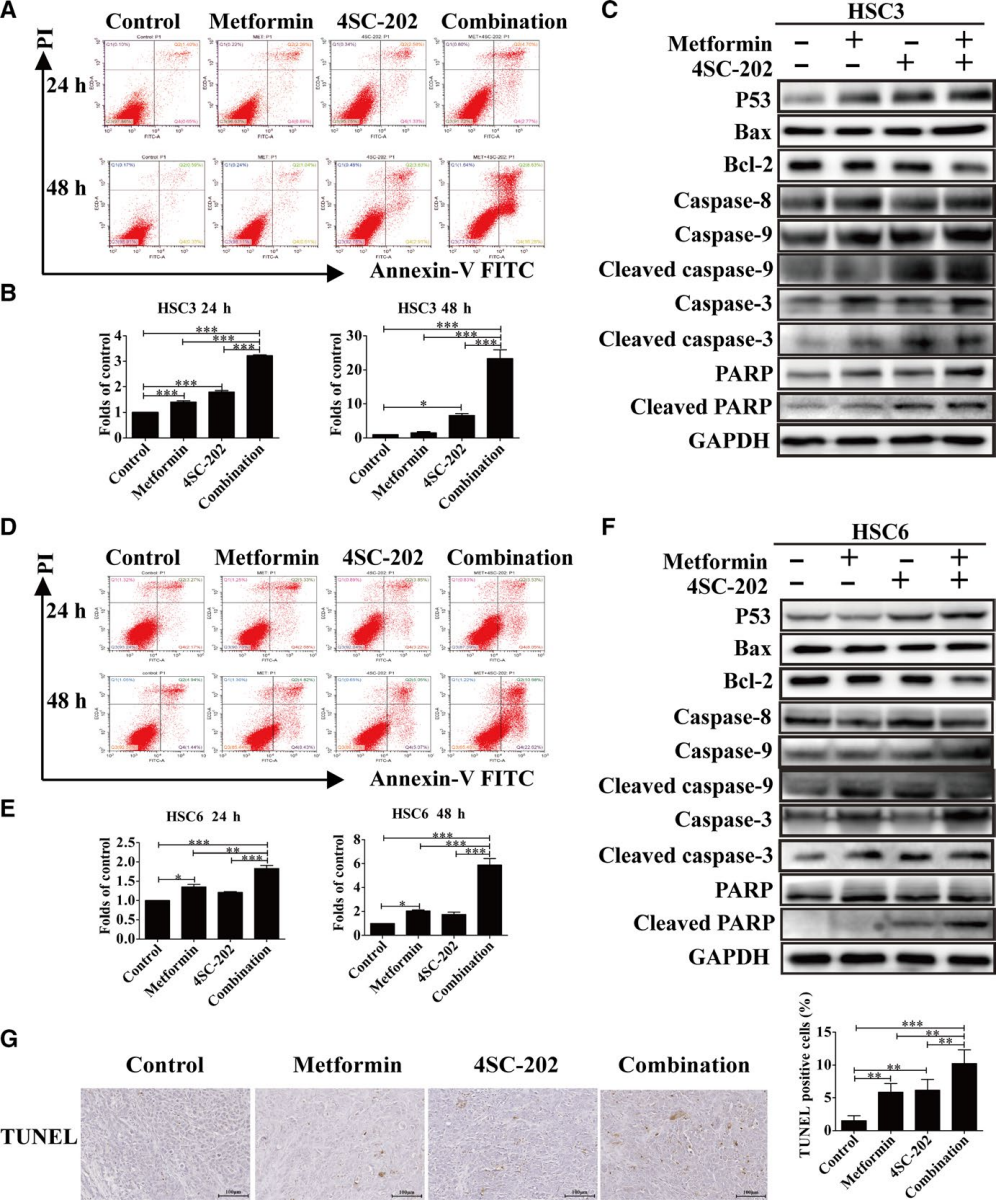
Metformin and 4SC‐202 synergistically promoted intrinsic cell apoptosis in OSCC. HSC3 or HSC6 cells were treated with metformin (16 mmol/L) or/and 4SC‐202 (0.4 μmol/L) for 24 and 48 hours. (A and D) Representative images of apoptosis evaluated with Annexin V‐FITC/PI staining in HSC3 or HSC6 cells. (B and E) The folds of change of apoptosis rates in HSC3 or HSC6 cells. (C and F) Expression levels of P53, Bax, Bcl‐2, cleaved caspase‐9, cleaved caspase‐3, cleaved PARP, and caspase‐8 were detected by western blot analysis in HSC3 or HSC6 cells after being treated for 24 hours. GAPDH was used as an internal control. (G) The apoptotic cells of tumor xenograft were determined by TUNEL staining under metformin or/and 4SC‐202 treatment. Data were shown as the means ± SD from three independent experiments. ^*^ *P* < 0.05, ^**^ *P* < 0.01, ^***^ *P* < 0.001 vs control (one‐way ANOVA)

Subsequently, we investigated whether apoptosis was induced by metformin and 4SC‐202 in OSCC. Flow cytometry analysis showed that both metformin and 4SC‐202 increased the number of apoptotic cells significantly compared to untreated cells. Intriguingly, the combination of metformin and 4SC‐202 had the maximum number of apoptotic cells (*P* < 0.05), as HSC3 apoptosis rate increased by 3.22 ± 0.05‐ and 23.32 ± 3.71‐fold after 24 and 48 hours (Figure 2A,B), and HSC6 apoptosis rate increased by 1.82 ± 0.12‐ and 5.88 ± 0.76‐fold after 24 and 48 hours (Figure 2D,E). Additionally, these results were further confirmed by western blot analysis. Combined treatment significantly increased the level of the intrinsic apoptosis makers such as P53, Bax, cleaved caspase‐9, cleaved caspase‐3, cleaved PARP, and decreased the protein level of Bcl‐2 in both HSC3 and HSC6 cells, compared to single treatment (Figure 2C,F). However, the extrinsic apoptosis key component caspase‐8 showed no significant alteration after either drug treatment. In addition, TUNEL staining of tumor xenograft model further confirmed that metformin or/and 4SC‐202 treatment increased cell apoptosis rate, and the combined treatment (10.19 ± 1.84%) had the most dramatical increase (*P* < 0.01) compared to control (1.49 ± 0.68%) (Figure 2G). Thus, metformin and 4SC‐202 synergistically promote intrinsic apoptosis in OSCC in vitro and in vivo.

### 3.3 Combined metformin and 4SC‐202 treatment inhibited oral carcinogenesis in vivo

**Figure 3:**
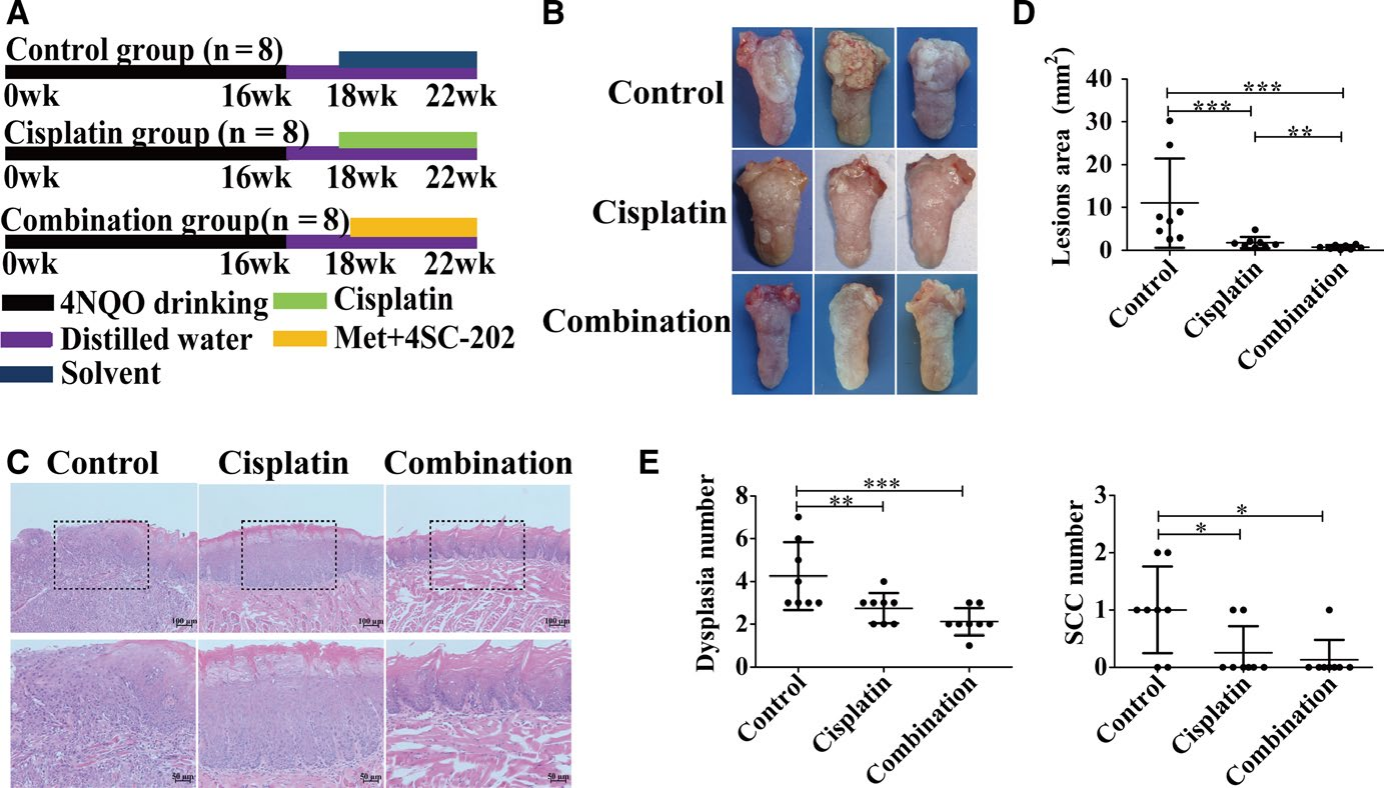
Combined metformin and 4SC‐202 treatment inhibited carcinogenesis of oral carcinoma in vivo. (A) Schematic diagrams showed the experimental strategies for 4NQO mice model. Met + 4SC‐202 means metformin plus 4SC‐202. (B) Representative figures of tongue in different treatment groups. (C) Representative image of H&E staining of OSCC in different groups. (D) Quantification of lesion areas from different treatment groups. (E) Quantification of dysplasia and SCC numbers in different treatment groups. Data were shown as the means ± SD from three independent experiments. ^*^ *P* < 0.05, ^**^ *P* < 0.01, ^***^ *P* < 0.001 vs control (Kruskal‐Wallis test)

4NQO‐induced mice OSCC model was utilized to investigate the effect of metformin and 4SC‐202 treatment on the development of oral cancer (Figure 3A). Our results revealed that metformin plus 4SC‐202 or cisplatin reduced the lesions area of tongue (*P* < 0.001) compared to the control, and metformin plus 4SC‐202 had a stronger inhibitory effect (*P* < 0.01) vs cispaltin (Figure 3B‐D). Importantly, histological results indicated that the number of dysplasia and squamous cell carcinoma (SCC) decreased significantly (*P* < 0.05) under metformin plus 4SC‐202 or cisplatin treatment (Figure 3E). Interestingly, there was no significant body weight loss both in combination group and cisplatin group compared to the control group, while the cisplatin group tended to had more weight loss (*P* < 0.05) vs combination group (Figure S1C). Meanwhile, H&E staining indicated that there was no obvious histopathological alteration in the liver and kidney tissues after treatment with drugs (Figure S1D). Overall, these results indicated that metformin and 4SC‐202 suppressed the progression of oral carcinoma in 4NQO induced mouse model.

### 3.4 Metformin and 4SC‐202 combination promoted ΔNp63 degradation via ubiquitination

**Figure 4:**
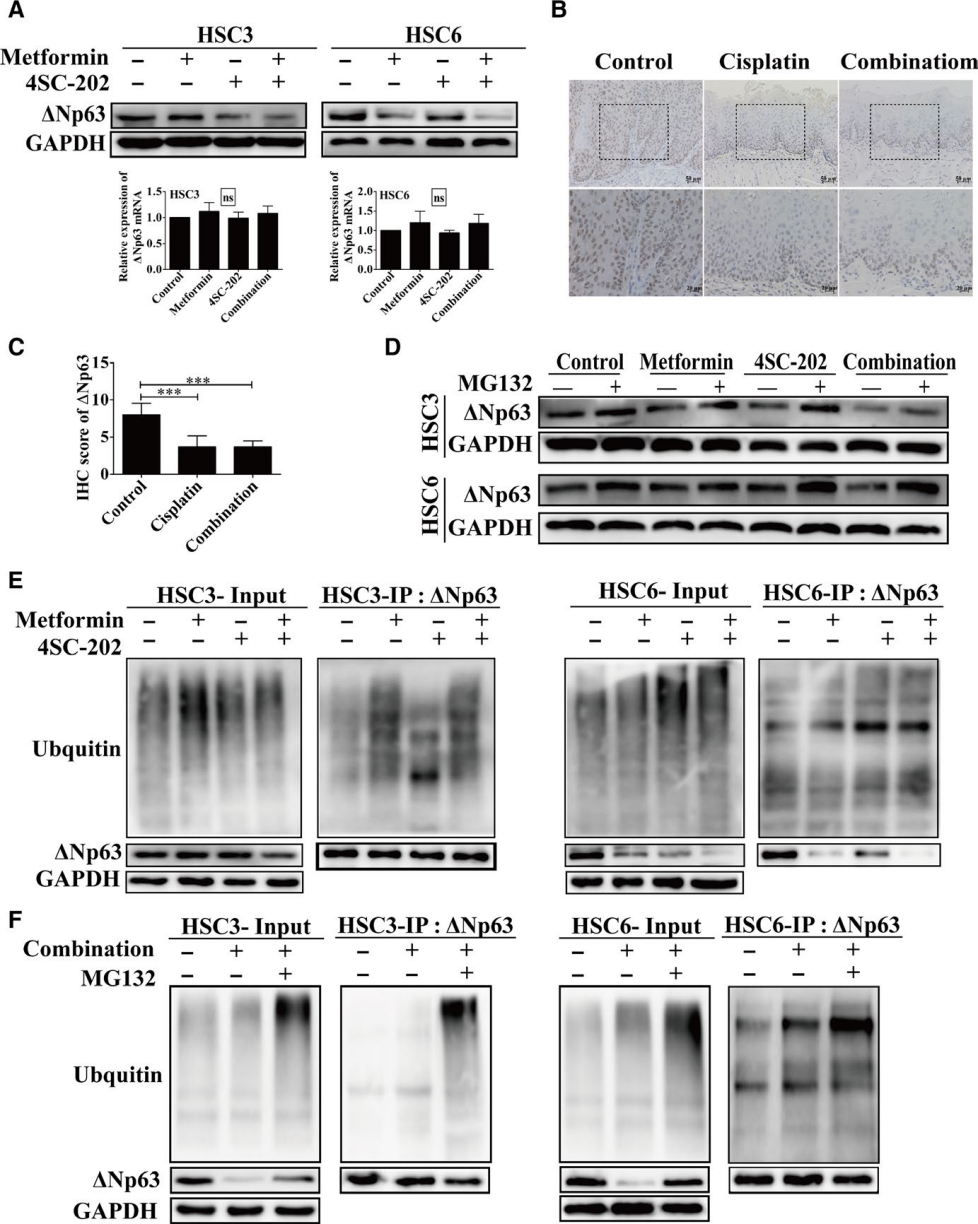
Metformin and 4SC‐202 combination reduced expression level of ΔNp63. (A) The protein or the mRNA level of ΔNp63 were determined by western blot or RT‐PCR under different conditions in HSC3 or HSC6 cells, respectively. (B) Representative images of IHC staining of ΔNp63 protein in 4NQO mice model. (C) The score of IHC staining of ΔNp63 protein in 4NQO mice model. (D) The protein level of ΔNp63 with or without MG132 (5 μmol/L) treatment for 6 hours was revealed by western blot. (E) Co‐IP was performed to determine the ubiquitination level of ΔNp63 in OSCC with metformin or/and 4SC‐202. (F) Co‐IP was performed to determine the ubiquitination level of ΔNp63 in OSCC under metformin plus 4SC‐202 with or without MG132. GAPDH was used as an internal control. Data were shown as the means ± SD for three independent experiments. ^***^ *P* < 0.001 compared to control (Kruskal‐Wallis test)

Overexpression of ΔNp63 isoforms of TP63 is observed in the majority of HNSCCs.^30^ ΔNp63 acts as an oncogene which suppresses apoptosis but sustains proliferation, and aberrant expression of ΔNp63 is associated with the poor prognosis of OSCC patients.^30^, ^31^, ^32^ Here, we detected the expression of ΔNp63 by western blot and RT‐PCR after treating with metformin or/and 4SC‐202. The protein level of ΔNp63 was decreased remarkably under 4SC‐202 or metformin administration, especially in combined group, but the mRNA level of ΔNp63 remained unperturbed (*P* > 0.05) (Figure 4A).Similarly, metformin plus 4SC‐202 treatment reduced the level of ΔNp63 (*P* < 0.001) in vivo (Figure 4B,C). Besides, the results revealed that cisplatin could also reduce ΔNp63 in vivo as well as metformin and 4SC‐202 (Figure 4B). Moreover, cisplatin was found to reduce the mRNA and protein level of ΔNp63 in HSC3 and HSC6 (Figure S2). However, compared to metformin plus 4SC‐202, cisplatin led to less decrease in ΔNp63 protein (Figure S2B). Subsequently, we examined whether ΔNp63 protein stability was regulated by proteasome‐mediated degradation by using the proteasome inhibitor MG132. As the results showed, the decreased of ΔNp63 protein under metformin or/and 4SC‐202 treatment was attenuated by MG132 administration (Figure 4D). Furthermore, the ubiquitination level of ΔNp63 with metformin or/and 4SC‐202 treatment was determined by Co‐immunoprecipitation (Co‐IP) analysis. Metformin or 4SC‐202 alone was observed to increase the ubiquitination level of ΔNp63, while the combination had the maximum increase (Figure 4E). Moreover, the ubiquitination level of ΔNp63 was increased in cells with MG132 under metformin plus 4SC‐202 treatment. (Figure 4F). Taken together, these data indicated that metformin and 4SC‐202 increased ΔNp63 protein ubiquitination and subsequently decreased its stability and protein level.

### 3.5 ΔNp63 mediated the apoptosis‐promoting effects of metformin and 4SC‐202

**Figure 5:**
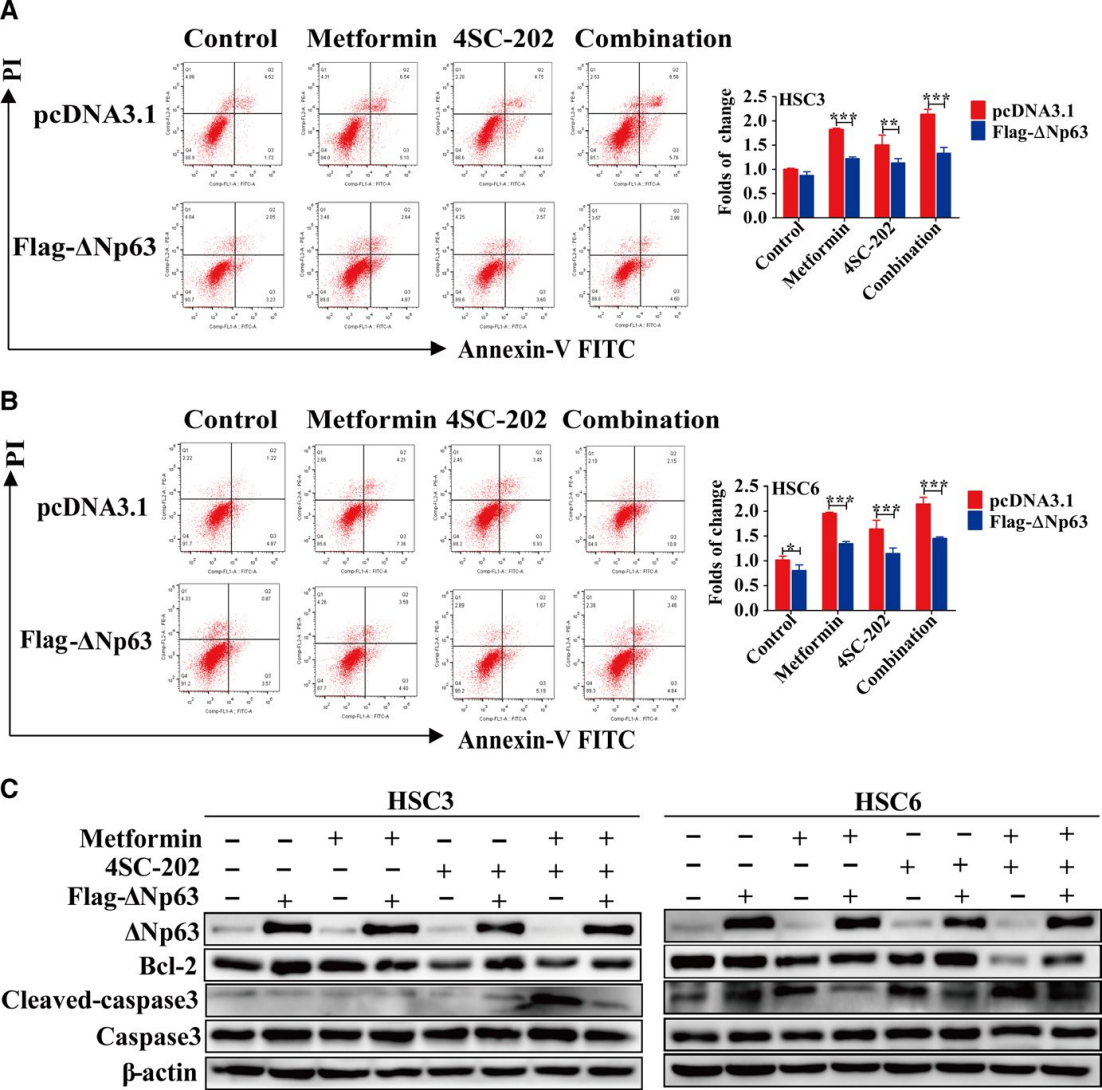
A ΔNp63 mediated the antitumor effects of combination of metformin and 4SC‐202. HSC3 or HSC6 cells were treatment with metformin (16 mmol/L) or/and 4SC‐202 (0.4 μmol/L) for 24 hours after overexpression of ΔNp63 for 24 hours. (A‐B) The apoptosis of HSC3 or HSC6 cells was evaluated by Annexin V‐FITC/PI staining. (C) Expression levels of Bcl‐2 and cleaved caspase‐3 were detected by western blot analysis. β‐actin was used as an internal control. Data were shown as the means ± SD for three independent experiments. ^*^ *P* < 0.05, ^**^ *P* < 0.01, ^***^ *P* < 0. 01 vs control (pcDNA3.1) (one‐way ANOVA).

To explore the role of ΔNp63 in antitumor effects of metformin and 4SC‐202, ΔNp63 was overexpressed or knockdown under metformin or/and 4SC‐202 treatment. Annexin V‐FITC/PI double‐staining was performed to determine the apoptosis rate. As the result revealed, with ΔNp63 overexpression, the apoptosis rate decreased from 1.82 ± 0.03‐ to 1.23 ± 0.03‐fold under metformin treatment, from 1.50 ± 0.17‐ to 1.34 ± 0.07‐fold under 4SC‐202 treatment, and from 2.14 ± 0.09‐ to 1.33 ± 0.10‐fold under combination of them (*P* < 0.01) in HSC3 cells (Figure 5A). Similarly, the apoptosis rate decreased from 1.96 ± 0.02‐ to 1.34 ± 0.04‐fold under metformin treatment, from 1.64 ± 0.15‐ to 1.14 ± 0.09‐fold under 4SC‐202 treatment, and from 2.14 ± 0.11‐ to 1.45 ± 0.02‐fold under combination of them (*P* < 0.05) in HSC6 cells (Figure 5B). Subsequently, western blot analysis showed that ΔNp63 lowered the expression level of cleaved caspase‐3 and increased the expression level of Bcl‐2 in HSC3 and HSC6 cells under 4SC‐202 or/and metformin treatment (Figure 5C,D). Moreover, knockdown of ΔNp63 increased the apoptosis rate of HSC3 and HSC6 (*P* < 0.01), promoted the expression of cleaved‐caspase‐3 and reduced the expression of Bcl‐2 (Figure S3).

## 4 DISCUSSION

In this study, the efficacy of metformin and 4SC‐202 combination in OSCC in vitro and in vivo was evaluated. Our results indicated that metformin and 4SC‐202 synergistically suppressed the growth and promoted the intrinsic apoptosis in OSCC. In addition, combined 4SC‐202 and metformin inhibited oral carcinogenesis in vivo. Importantly, metformin or/and 4SC‐202 triggered apoptosis of OSCC through accelerating the degradation of ΔNp63. These findings highlighted that this combination could serve as a potential therapeutic schemes for OSCC.

Current chemotherapy treatments for OSCC are not satisfactory for drug resistance and side effects, which has become a challenge in the clinics. As a promising candidate to overcome these problems in cancer therapy, combination of chemotherapeutics could take the advantage of each drug and lower the dose and toxicity. Here, we evaluated the effects of metformin, 4SC‐202, and their combination in OSCC, and showed dose‐ and time‐dependent growth inhibitory effect of 4SC‐202 and metformin. Notably, the combination of metformin and 4SC‐202 showed synergistic growth inhibitory effects in OSCC cells. We applied a relatively low dose of metformin (16 mmol/L, IC_30‐40_) and 4SC‐202 (0.4 μmol/L, IC_30‐40_) which inhibited tumor growth effectively. For xenograft or 4NQO mouse model, we chose a relatively low dose of metformin (100 mg/kg) and 4SC‐202 (80 mg/kg) to lower possible toxicity. The tumor volumes and weights of nude mice that received metformin and 4SC‐202 were smaller compared to control group without apparent body weight loss and liver and kidney impairment, indicating that metformin and 4SC‐202 synergistically inhibited tumor growth and were relatively safe in vivo We were aware of the deficiency of using only one cell line HSC6, and we had tried the other cell line HSC3 in nude mice; however, we found that the tumors were easily to break and form ulceration, which made it hard to measure volume and weight as the ulceration usually caused loss of tumor cells. Meanwhile, 4NQO‐induced mouse oral carcinogenesis further confirmed the inhibitory effect of combined treatment of metformin and 4SC‐202 in vivo, and it may work better than traditional medicine cisplatin. Collectively, our results indicated that metformin and 4SC‐202 synergistically suppressed tumor growth in vitro and in vivo.

In most cases, anticancer therapies eventually result in activation of apoptosis. In mammals, there are two major apoptotic pathways, the extrinsic pathway (death receptor‐mediated pathway) and the intrinsic pathway (mitochondrial‐mediated pathway). Activation of caspases usually are initiated from two main entry points at the death receptor (extrinsic pathway) or at the mitochondria (mitochondrial‐mediated pathway).^33^ In previous works, metformin was found to induce intrinsic apoptotic pathway in oral cancer cells.^34^, ^35^ However, the effects of 4SC‐202 in OSCC remains unclear. In hepatocellular carcinoma cells, intrinsic apoptotic pathway was activated under 4SC‐202 treatment.^26^ Our results displayed that combined metformin and 4SC‐202 treatment synergistically induced cell apoptosis compared to single treatment in OSCC. The mitochondrial pathway proteins in the combination group changed significantly, while the key extrinsic apoptotic component caspase‐8 showed no significant change, suggesting that intrinsic apoptotic pathway was regulated by the combination treatment. Metformin or/and 4SC‐202 promoted the expression of P53 and Bax but reduced the expression of Bcl‐2, and metformin plus 4SC‐202 had the most dramatical effects. In addition, previous works showed that P53 negatively regulated Bcl‐2 and positively regulated Bax by directly binding to its promoter;^36^, ^37^ therefore, the decrease level of Bcl‐2 and the increase level of Bax may be the result of increased level of P53. Metformin was found to activate AMPK signaling,^21^, ^38^ and AMPK could phosphorylate SIRT1 or MDMX to stabilize and activate P53.^39^, ^40^ HDACis were found to activate P53 and promoted P53 acetylation which result in P53 stabilization and activation.^41^, ^42^, ^43^ Thus, in OSCC cells, we speculated that metformin and 4SC‐202 promoted the expression of P53 through AMPK and promotion of P53 deacetylation. Moreover, the apoptosis‐promoting effects were further confirmed in xenograft model. Overall, our results indicated metformin or/and 4SC‐202 triggered intrinsic apoptosis of OSCC cells in vitro and in vivo.

Ubiquitination is among the most common forms of posttranslational protein modification. Proteins modified with ubiquitin, a small 8.5 kDa protein, are targeted for degradation by the proteasome.^44^, ^45^ This process is executed by three classes of enzymes designated E‐1, E‐2, and E‐3.^44^ E‐1 activation enzymes activate ubiquitin in an ATP‐dependent manner, attaching it to a cysteine residue of an E‐2 conjugation enzyme.^45^ The E‐2 conjugation enzyme coordinates with an E‐3 ligase enzyme to attach ubiquitin to a lysine residue of a target substrate.^46^ E‐3 ligases are substrate‐specific and recognizes the target protein.^45^ Our results revealed that ΔNp63 protein level decreased without significant alteration in mRNA, indicating ΔNp63 might be regulated at the posttranslational level such as ubiquitination. ΔNp63 is targeted by multiple E‐3 ligases such as WWP1, HDM2, FBXW7, Itch, and Pirh2, for ubiquitination and proteasome‐mediated degradation, which acts as new key regulators of the P63 protein.^47^, ^48^ In our results, MG132 attenuated the downregulation of ΔNp63 and ubiquitination level of ΔNp63 increased significantly when cells treated with metformin or/and 4SC‐202 in OSCC. Furthermore, we examined the ubiquitination level of ΔNp63 under metformin and 4SC‐202 treatment with or without MG132, as the results revealed, MG132 treatment increased the ubiquitination level of ΔNp63 under metformin and 4SC‐202 treatment. Taken together, metformin and 4SC‐202 accelerated ubiquitination and proteasome‐mediated degradation of ΔNp63.

Previous studies had identified ΔNp63 as an oncogene which suppresses apoptosis and sustains proliferation, and aberrant expression of ΔNp63 was associated with the poor prognosis of patients with OSCC.^30^, ^31^, ^32^, ^49^ ΔNp63 acts primarily in dominant‐negative of P63, while the structure and function of full‐length TA (transactivation) isoform of P63 has similarity to wild‐type P53, the ΔNp63 acts primarily against nearly all family members of P53.^50^ Overexpression of ΔNp63 isoforms is observed in the majority of HNSCCs.^30^ In addition, high expression of ΔNp63 contributes to chemoresistance.^51^, ^52^ As our results showed, ΔNp63 protein level decreased after metformin or/and 4SC‐202 treatment, and combined treatment led to significant decrease of ΔNp63 compared to single treatment. The ΔNp63‐reducing effect of metformin and 4SC‐202 treatment was further confirmed in 4NQO mice model. Intriguingly, we found that cisplatin treatment also led to the decrease of ΔNp63, which was observed in vitro and in vivo. As both mRNA and protein level alterations were observed, we speculate that cisplatin may reduce ΔNp63 through transcriptional regulation which was different from metformin and 4SC‐202. In addition, compared to cisplatin, metformin plus 4SC‐202 led to greater decrease of ΔNp63 protein in OSCC, indicating that metformin plus 4SC‐202 had stronger inhibition effect on ΔNp63. Notably, ΔNp63 overexpression eliminated the proapoptotic effects of metformin and 4SC‐202, while knockdown of ΔNp63 facilitated the proapoptotic effects. Collectively, ΔNp63 is a major target of metformin and 4SC‐202 in their facilitation of apoptosis.

In conclusion, combined treatment of metformin and 4SC‐202 synergistically inhibit cancer cell growth and induce intrinsic cell apoptosis through increasing ΔNp63 ubiquitination and degradation in vitro and in vivo. Combined metformin and 4SC‐202 treatment could be a promising therapeutic strategy for OSCC.

## CONFLICT OF INTEREST

The authors have no conflict of interest.
