# Supplementary material for: Metformin and 4SC‐202 synergistically promote intrinsic cell apoptosis by accelerating ΔNp63 ubiquitination and degradation in oral squamous cell carcinoma
Source: Cancer Med. 2019 Apr 25;8(7):3479–90. doi: 10.1002/cam4.2206 (PMC6601594; doi:10.1002/cam4.2206)
Supplement: Supplementary file 1 [file CAM4-8-3479-s001.docx]

**Figure S1**

| 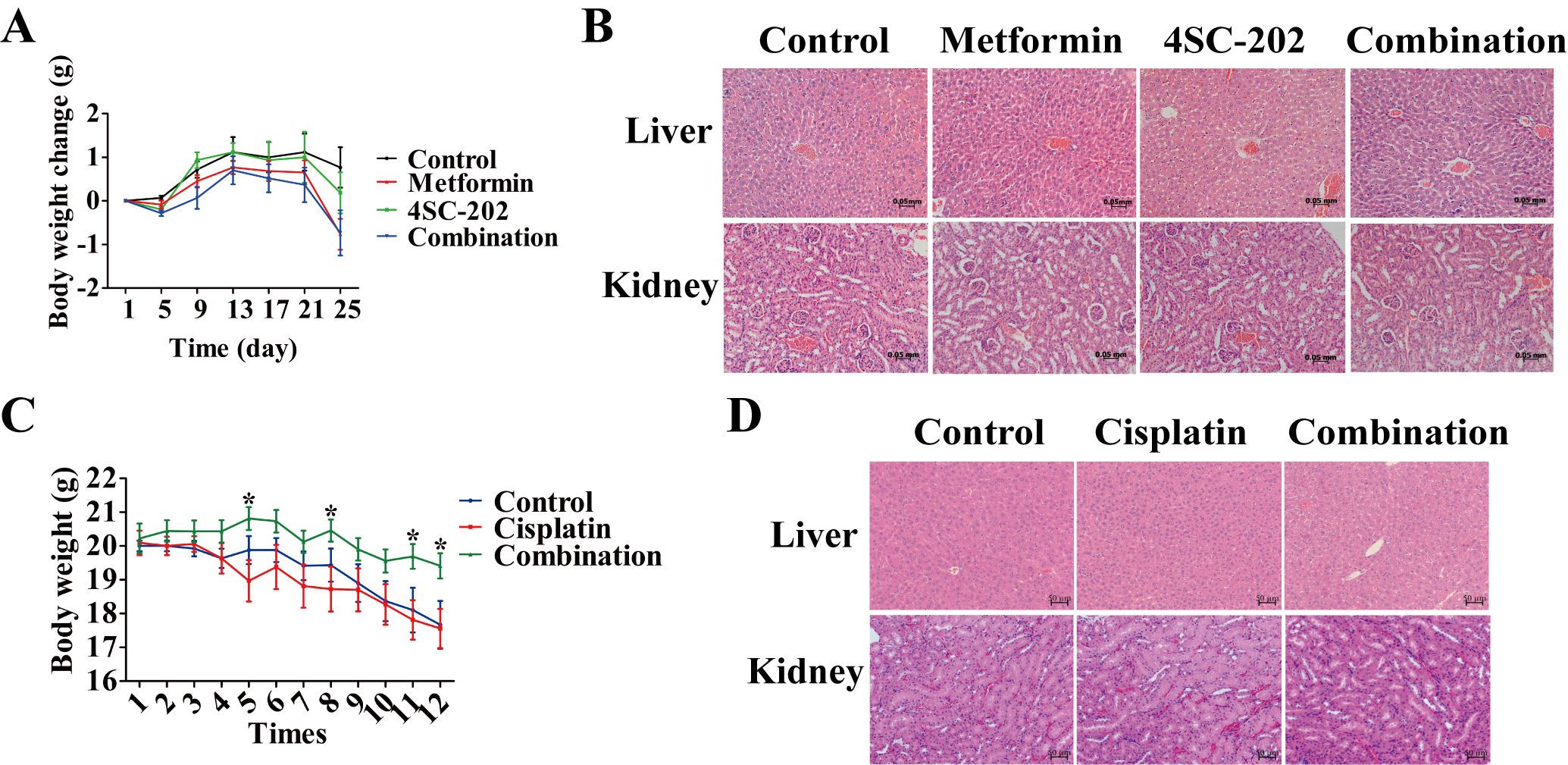 |
| --- |

**Figure S1 The body weight and histomorphology of liver and kidney of mice.** A&C: The body weight of nude mice or 4NQO mice in different time under different conditions. B&D: Representative images of H&E staining of liver and kidney of nude mice or 4NQO mice under different conditions. Data were shown as the means ± SD for three independent experiment. **P* < 0.05 vs cisplatin (one-way ANOVA).
