# Supplementary material for: Metformin and 4SC‐202 synergistically promote intrinsic cell apoptosis by accelerating ΔNp63 ubiquitination and degradation in oral squamous cell carcinoma
Source: Cancer Med. 2019 Apr 25;8(7):3479–90. doi: 10.1002/cam4.2206 (PMC6601594; doi:10.1002/cam4.2206)
Supplement: Supplementary file 2 [file CAM4-8-3479-s002.docx]

**Figure S2**

| \| 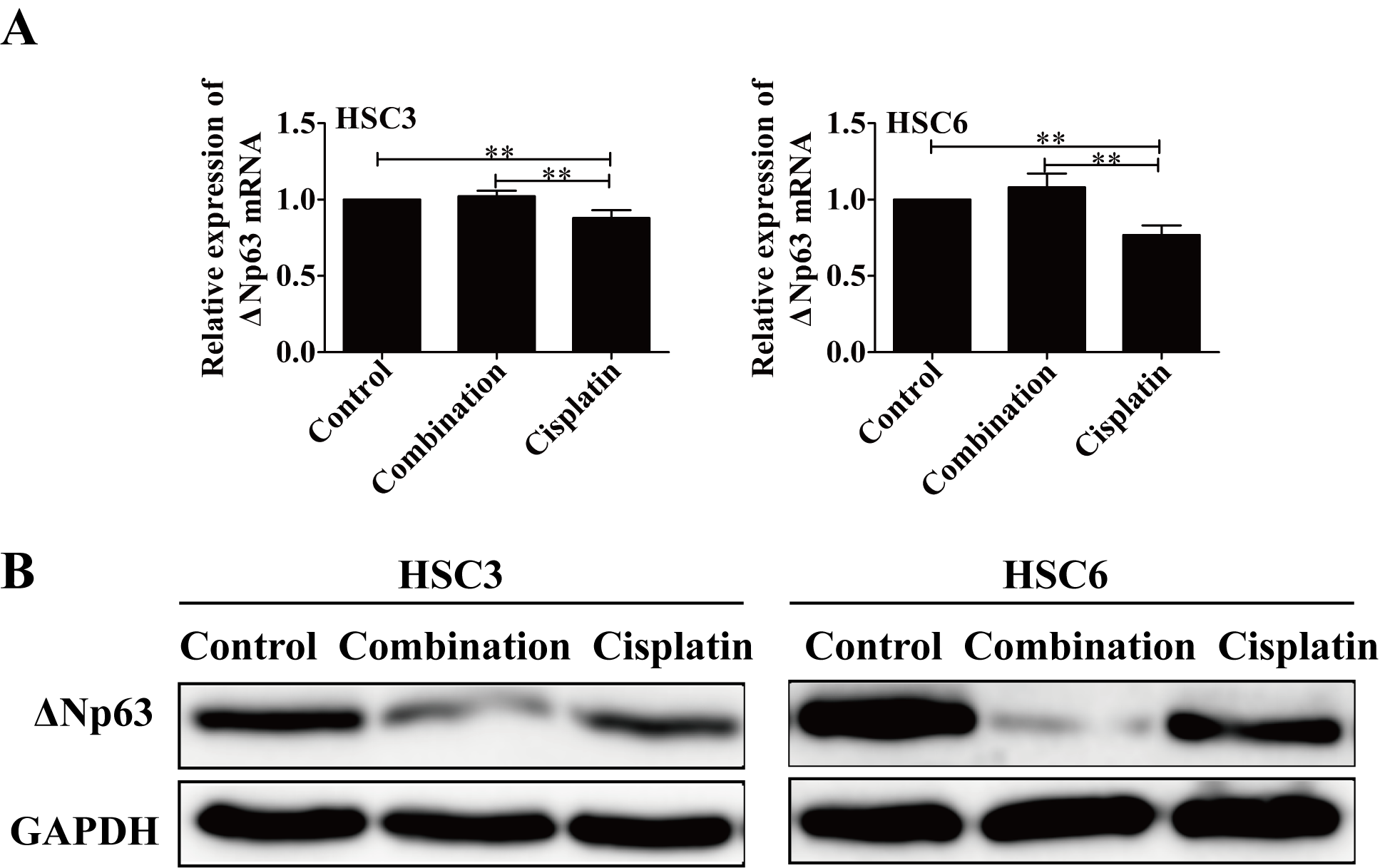 \| \| --- \| |
| --- | --- |
|  |

**Figure S2 The ΔNp63 level in HSC3 and HSC6 cells under cisplatin treatment.** HSC3 or HSC6 cells were under cisplatin (12 μM) or metformin (16 mM) plus 4SC-202 (0.4 μM) treatment for 24 h. A: The mRNA level of ΔNp63 were determined by RT-PCR under different conditions in HSC3 or HSC6 cells, respectively. Data were shown as the means ± SD for three independent experiment. ***P* < 0.01 vs control. B: The protein level of ΔNp63 were determined by western blot under different conditions in HSC3 or HSC6 cells, respectively.
