# Supplementary material for: Metformin and 4SC‐202 synergistically promote intrinsic cell apoptosis by accelerating ΔNp63 ubiquitination and degradation in oral squamous cell carcinoma
Source: Cancer Med. 2019 Apr 25;8(7):3479–90. doi: 10.1002/cam4.2206 (PMC6601594; doi:10.1002/cam4.2206)
Supplement: Supplementary file 3 [file CAM4-8-3479-s003.docx]

**Figure S3**

| 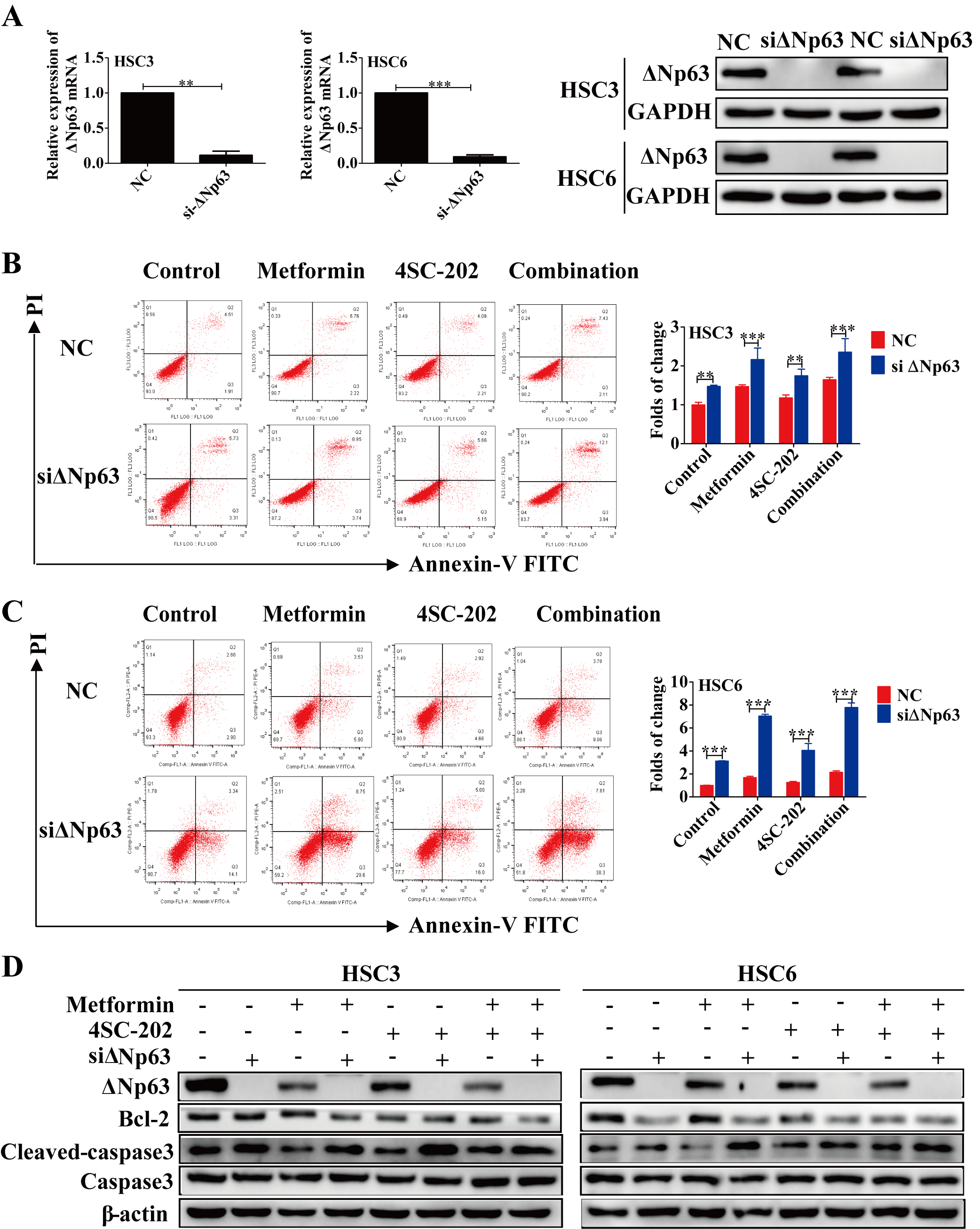 |
| --- |

**Figure S3 ΔNp63 knockdown facilitated the apoptosis effects of metformin and 4SC-202.** HSC3 or HSC6 cells were treatment with metformin (16 mM) or/and 4SC-202 (0.4 μM) for 24 h after transfected with ΔNp63 siRNA for 24 h. A: The knockdown efficiency of siΔNp63 was validated by western blot and RT-PCR in HSC3 or HSC6 cells. ***P* < 0.01 and ****P* < 0.001 vs NC (Student's t-test). B-C: The apoptosis of HSC3 or HSC6 cells were evaluated by Annexin V-FITC/PI staining. D: Expression levels of Bcl-2 and cleaved caspase-3 were detected by western blot. Data were shown as the means ± SD for three independent experiment. ***P* < 0.01 and ****P* < 0.001 vs control (NC) (one-way ANOVA).
