# Supplementary material for: Metformin and 4SC‐202 synergistically promote intrinsic cell apoptosis by accelerating ΔNp63 ubiquitination and degradation in oral squamous cell carcinoma
Source: Cancer Med. 2019 Apr 25;8(7):3479–90. doi: 10.1002/cam4.2206 (PMC6601594; doi:10.1002/cam4.2206)
Supplement: Supplementary file 4 [file CAM4-8-3479-s004.docx]

**Table S1** Reagents and primary antibodies information

| Reagent | Company | Catalog No. | Application |
| --- | --- | --- | --- |
| 4SC-202 | Selleckchem, Houston, TX, USA | S7555 | Cell culture, 0.4μM;  Animal experiment, 80mg/kg |
| Metformin | Sigma-Aldrich, Merck KGaA, Darmstadt, Germany | PHR1084 | Cell culture,16mM;  Animal experiment, 100mg/kg |
| Cisplatin | Selleckchem, Houston, TX, USA | S1166 | Animal experiment, 1mg/kg |
| 4NQO | Sigma-Aldrich, Merck KGaA, Darmstadt, Germany | N8141 | Animal experiment, 50μg/ml |
| MG132 | Selleckchem, Houston, TX, USA | S2619 | Cell culture, |
| Bcl-2 antibody | Affinity, Cincinnati, OH, USA | AF6139 | WB, 1:1000 |
| P53 antibody | Affinity, Cincinnati, OH, USA | AF0879 | WB, 1:1000 |
| β-actin antibody | Affinity, Cincinnati, OH, USA | AF7018 | WB, 1:1000 |
| Cleaved caspase-3 antibody | Affinity, Cincinnati, OH, USA | AF7022 | WB, 1:1000 |
| Caspase-3 antibody | Cell Signaling, Beverly, MA, USA | 9665 | WB, 1:1000 |
| Bax antibody | Cell Signaling, Beverly, MA, USA | 2772 | WB, 1:1000 |
| Caspase-8 antibody | Cell Signaling, Beverly, MA, USA | 4790 | WB, 1:1000 |
| Cleaved caspase-9 antibody | Cell Signaling, Beverly, MA, USA | 52873 | WB, 1:1000 |
| Caspase-9 antibody | Cell Signaling, Beverly, MA, USA | 9502 | WB, 1:1000 |
| PARP | Cell Signaling, Beverly, MA, USA | 9532 | WB, 1:1000 |
| cleaved-PARP | Cell Signaling, Beverly, MA, USA | 5625 | WB, 1:1000 |
| GAPDH | Cell Signaling, Beverly, MA, USA | 5174 | WB, 1:1000 |
| ΔNp63 antibody | Biolegend, San Diego, CA, USA | 619002 | WB, 1:1000;  IHC, 1:200 |
| Ubiquitin antibody | ABclonal, Harrogate, HG2,UK | A3207 | WB, 1:1000 |

Note：

IHC: Immunohistochemistry

WB: Western blot
